# Electrically Stimulated Antagonist Muscle Contraction Increased Muscle Mass and Bone Mineral Density of One Astronaut - Initial Verification on the International Space Station

**DOI:** 10.1371/journal.pone.0134736

**Published:** 2015-08-21

**Authors:** Naoto Shiba, Hiroo Matsuse, Yoshio Takano, Kazuhiro Yoshimitsu, Masayuki Omoto, Ryuki Hashida, Yoshihiko Tagawa, Tomohisa Inada, Shin Yamada, Hiroshi Ohshima

**Affiliations:** 1 Department of Orthopedics, Kurume University School of Medicine, Kurume, Fukuoka, Japan; 2 Division of Rehabilitation, Kurume University School of Medicine, Kurume, Fukuoka, Japan; 3 Division of Physical Therapy, Fukuoka International University of Health and Welfare, Okawa city, Fukuoka 8318501, Japan; 4 Department of Mechanical and Control Engineering, Kyushu Institute of Technology, Kitakyushu, Fukuoka, Japan; 5 Space Environment Utilization Center, Japan Aerospace Exploration Agency, Tsukuba, Ibaraki, Japan; Medical University of Graz, AUSTRIA

## Abstract

**Background:**

Musculoskeletal atrophy is one of the major problems of extended periods of exposure to weightlessness such as on the International Space Station (ISS). We developed the Hybrid Training System (HTS) to maintain an astronaut’s musculoskeletal system using an electrically stimulated antagonist to resist the volitional contraction of the agonist instead of gravity. The present study assessed the system’s orbital operation capability and utility, as well as its preventative effect on an astronaut’s musculoskeletal atrophy.

**Methods:**

HTS was attached to the non-dominant arm of an astronaut staying on the ISS, and his dominant arm without HTS was established as the control (CTR). 10 sets of 10 reciprocal elbow curls were one training session, and 12 total sessions of training (3 times per week for 4 weeks) were performed. Pre and post flight ground based evaluations were performed by Biodex (muscle performance), MRI (muscle volume), and DXA (BMD, lean [muscle] mass, fat mass). Pre and post training inflight evaluations were performed by a hand held dynamometer (muscle force) and a measuring tape (upper arm circumference).

**Results:**

The experiment was completed on schedule, and HTS functioned well without problems. Isokinetic elbow extension torque (Nm) changed -19.4% in HTS, and -21.7% in CTR. Isokinetic elbow flexion torque changed -23.7% in HTS, and there was no change in CTR. Total Work (Joule) of elbow extension changed -8.3% in HTS, and +0.3% in CTR. For elbow flexion it changed -23.3% in HTS and -32.6% in CTR. Average Power (Watts) of elbow extension changed +22.1% in HTS and -8.0% in CTR. For elbow flexion it changed -6.5% in HTS and -4.8% in CTR. Triceps muscle volume according to MRI changed +11.7% and that of biceps was +2.1% using HTS, however -0.1% and -0.4% respectively for CTR. BMD changed +4.6% in the HTS arm and -1.2% for CTR. Lean (muscle) mass of the arm changed only +10.6% in HTS. Fat mass changed -12.6% in HTS and -6.4% in CTR.

**Conclusions:**

These results showed the orbital operation capability and utility, and the preventive effect of HTS for an astronaut’s musculoskeletal atrophy. The initial flight data together with the ground data obtained so far will be utilized in the future planning of human space exploration.

## Introduction

It is well known that atrophy of the musculoskeletal system due to disuse occurs among astronauts. For instance, atrophy of the muscles and bones of astronauts in weightlessness is evident [1, 2]. Sarcopenia is widely used as one of the terms to express weakness and atrophy of the muscles, although it initially referred to atrophy due to aging. However this initial Sarcopenia has been classified as primary Sarcopenia, and all the other muscle weakness and atrophy has been included in secondary Sarcopenia by the Report of the European Working Group on Sarcopenia in Older People 2010 [3]. Disuse muscle atrophy is classified as secondary Sarcopenia, and we often experience it as a result of reduction of activity such as in the case of a clinically bed ridden patient. Zero gravity has been included as a cause of disuse and categorized as secondary Sarcopenia in this 2010 categorization.

A decrease in muscle volume was reported by 4–16%, and in strength was reported by 9–11% after 5–17 day short-term Space Shuttle flights [4–6]. 16–28 weeks stays on the Station Mir resulted in changes of 12–20% in volume [7]. It was also reported that volume of the gastrocnemius and soleus muscles decreased by 10% and by 15% respectively, and concomitant peak plantar flexor power decreased by 32% in nine subjects after about 6 months on the International Space Station (ISS) [8].

Astronauts usually exhibit a decrease in Bone Mineral Density (BMD) in the hip and lumbar spine by 1.0% to 1.5% per month [2,9,10]. This finding highlights the accelerated rate of BMD loss during spaceflight, contrasting remarkably with the typical age related rate of bone loss of 0.5% to 1.0% per year in older individuals on Earth [2]. The risk of fractures is of particular concern upon re-exposure to mechanical loading, such as during the exploration of an unknown planet or return to Earth’s full gravity field. Moreover, cumulative skeletal deconditioning could increase the risk of premature osteoporosis and of fractures later in life.

Such deteriorations of the musculoskeletal system are one of the main issues for a long term stay in space, and several countermeasures have been developed and their effectiveness reported [1,2,11–14]. Concerning these countermeasures, the evidence report of the Human Research Program of NASA described as follows [15]. A very simple exercise device which consisted of nylon cords rotating around a shaft within the cylinder was used on some Apollo missions. After the first manned mission to Skylab 2 (Skylab project was the first United States orbital space station), a commercial device, termed “Mini Gym,” modified extensively and designated “MK-I,” was available to transmit the force on the arms, trunk, and legs. Subsequently developed exercise devices provided loads by extension springs or bungee cords. In the early phase of the ISS program, the interim Resistive Exercise Device which was an elastomer-based resistive exercise hardware was used. A further developed version, called advanced Resistive Exercise Device (ARED) was delivered to ISS in 2010 and is in use now. ARED delivers adjustable loads of up to 600 pounds produced by vacuum canisters to enable a constant force and inertial flywheels to simulate the inertial loads that would be experienced using free weights at 1-G gravity. Thus, the development of countermeasures has advanced, ARED especially has provided good results. However, ARED occupies a large space and is difficult to use on a small spaceship which will be indispensable for missions to the moon, establishment of a lunar base, and interplanetary travel to Mars. Furthermore, there is only one ARED system in ISS, and thus the astronauts on the ISS can’t exercise at the same time. If the ARED breaks down, it is a severe problem.

**Fig 1:**
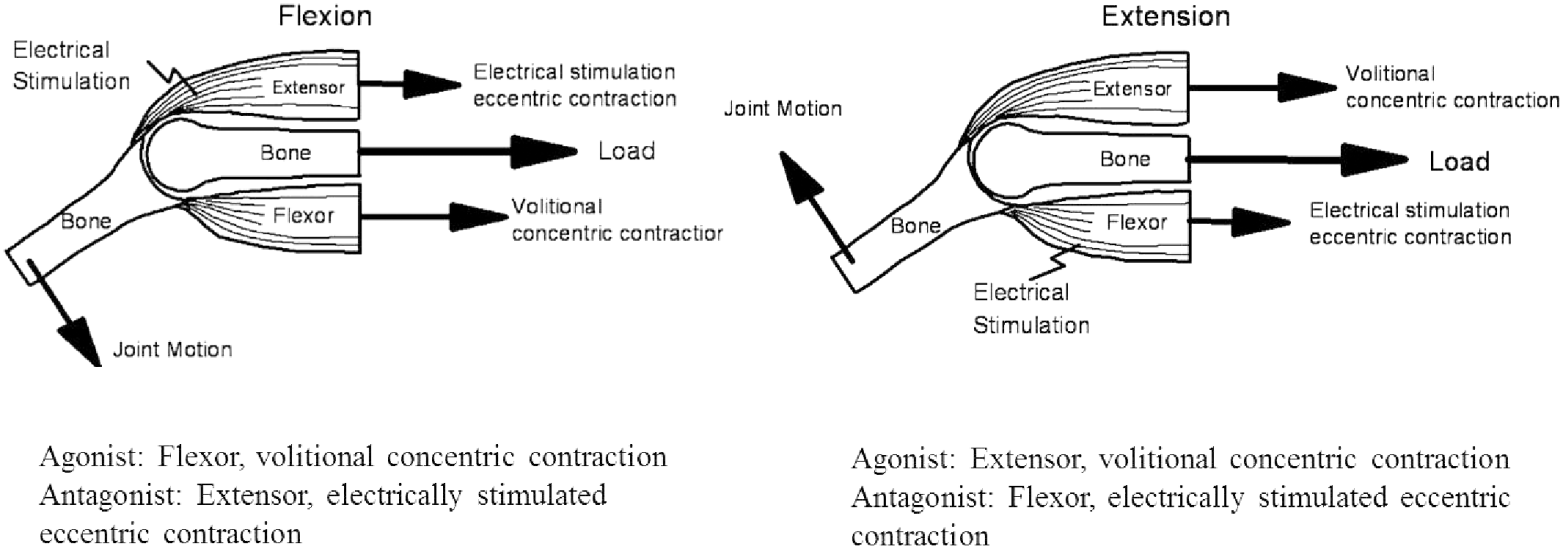
Schematic model of Hybrid training. Note that both the volitionally activated agonist and the electrically stimulated antagonist contract during joint motion. The result is that both muscles are exercised and that a longitudinal compressive load is placed on the bone.

We have developed a compact training device named “hybrid training system” (HTS) that is designed to maintain the musculoskeletal system of astronauts by using an electrically stimulated antagonist to resist the volitional contraction of agonist muscles (Fig 1) [16–22]. HTS, which utilizes electrically stimulated antagonist force as resistance to joint motion instead of gravity, causes electrically stimulated muscles to provide resistance to the motion of an antagonist muscle undergoing training. In other words, electrical stimulation is applied to the antagonist muscle during joint motion, the electrically stimulated antagonist muscle contracts eccentrically and its tension becomes a resistance to the agonist muscle. During HTS training, both flexion and extension muscles contract simultaneously, and longitudinal force load will be applied on the sandwiched bone by these contracting muscles. Multiple ground-based experiments [16–21] and a parabolic flight verification [22] have been encouraging as to the possibility of use by astronauts, but, thus far, evaluation in space has not been conducted.

In this experiment, as an initial verification on the ISS, HTS was used for one of an astronaut’s upper extremities (the non-dominant arm) for four weeks, and the resulting muscle strength, mass and BMD were compared to those of the non-HTS arm (the dominant arm) to examine its orbital operation capability and utility, as well as the preventive effect of HTS for musculoskeletal atrophy of an astronaut in weightlessness.

## Materials and Methods

### Ethics Statement

IRB of the Japanese Aerospace Exploration agency: JAXA and National Aeronautics and Space Administration: NASA approved the design of this study protocol. The subject was given oral and written explanations of the study involving the objective of the training method and its risks, and then signed consent forms for participation in this research. The subject was assured that he could discontinue if he wished. The individual of figure in this manuscript has given written informed consent (as outlined in PLOS consent form) to publish these case details.

### Subject

One of the ISS crew members was a candidate as the subject. The subject was allowed to perform daily operation and usual activities on the ISS including daily exercise and other experiments.

### Equipment

#### HTS

**Fig 2:**
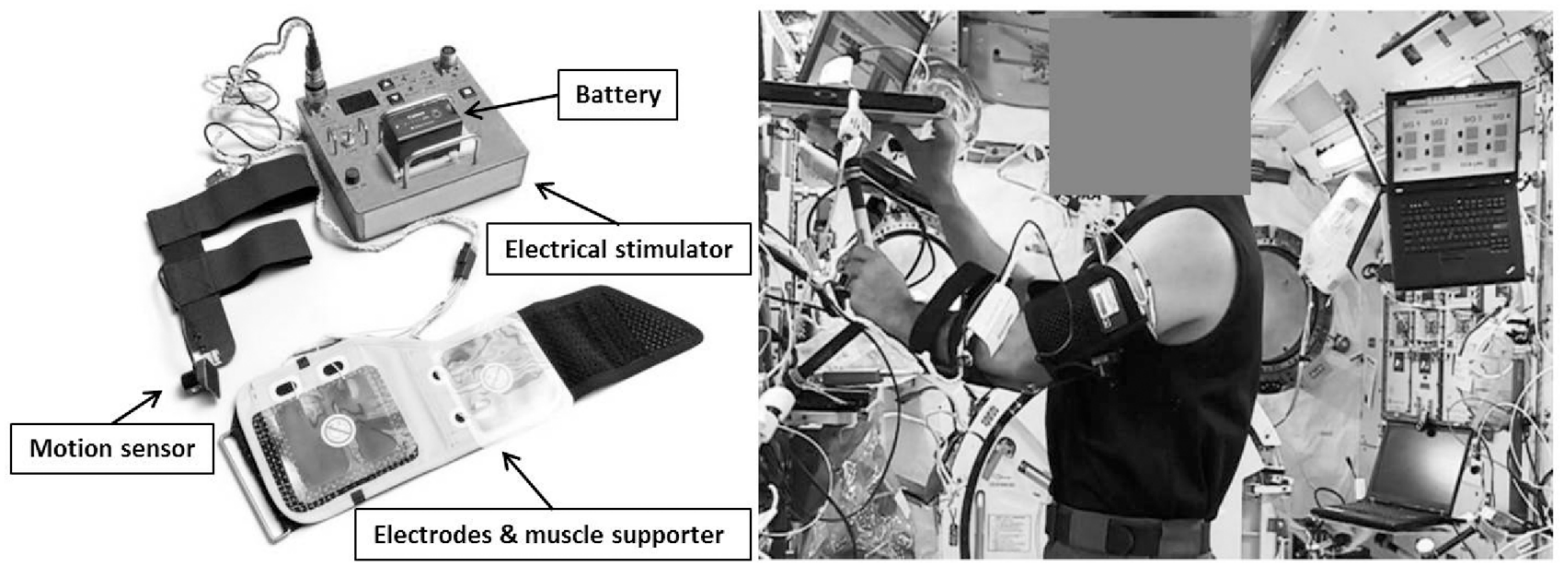
Hybrid training system device. The hybrid training system consisted of electric stimulator, battery, electrodes, motion sensor, and supporter. Electrodes were set inside of the supporter, and subject was easily able set them for himself properly. An astronaut attached the hybrid training system in the ISS for this experiment. (Courtesy by NASA and JAXA).

The system was basically the same as those used in previous ground based experiments [16–22], however it was designed and manufactured for use in the ISS under the criteria of NASA (Chiyoda Advance Solutions, CHIYODA CORPORATION, Yokohama, Japan). For example, the hardware of the ground model was changed so that harmful gas would not be generated in case of a fire, and the electromagnetic wave produced by the stimulator had no adverse effects on ISS. HTS consists of an electric stimulator, battery, electrodes, motion sensor, and a muscle supporter (Fig 2). The size and weight of the HTS device were 280 mm × 180 mm × 100 mm and 1650 g.

#### Stimulator

A constant voltage stimulator was used to prevent heat generation caused by impedance increase from deterioration or insufficient setting of the electrodes, and the restriction of electric current was also set to prevent overstimulation during decreasing impedance. The subject can set the stimulation intensity, and has the ability to determine overload for himself. The output of electric current is isolated by a transformer, and the DC component does not apply output to the human body. The maximum current of stimulation applied to the subject does not exceed 20mA in flight. The battery was a Canon BP-930 (Canon Inc. Tokyo Japan), which was already present on the ISS for a video camera. This battery was available for use, and lasts for 15 HTS training sessions when fully charged.

#### Electrode

Gel coated carbon electrodes (SEKISUI CHEMICAL CO.,LTD, Tokyo Japan) were attached over the motor points of the biceps and triceps muscles using a supporter. The supporter was designed so that the subject would be able to set the electrodes at the proper position easily for himself (GOLDWIN INC., Tokyo, Japan).

#### Motion sensor

Joint motion was detected by expansion and retraction of a wire connected to a rotary encoder (ME-20-P, MUOTH ENGINEERING INC., Tokyo, Japan). The resolution, maximal detection speed, and maximal acceleration of the wire-rotary encoder were 0.01 mm, 15 m / min, and 7.8 m / s^2^, respectively.

### Training protocol

#### Put on and set HTS

The electrodes were embedded in the supporter and attached over the motor points of the triceps and biceps muscles for the non-dominant upper arm only. Stimulation intensity was set at the level predetermined in a ground based adjustment 4 months before launch, and fixed for the ISS experiment. It was determined as 80% of maximal comfortable stimulation intensity as per previous ground based studies [16–22]. A constant stimulation intensity was used during the entire 4 week training period, and it was 21.0V on the triceps and 15.5V on the biceps muscle respectively.

#### HTS training

**Fig 3:**
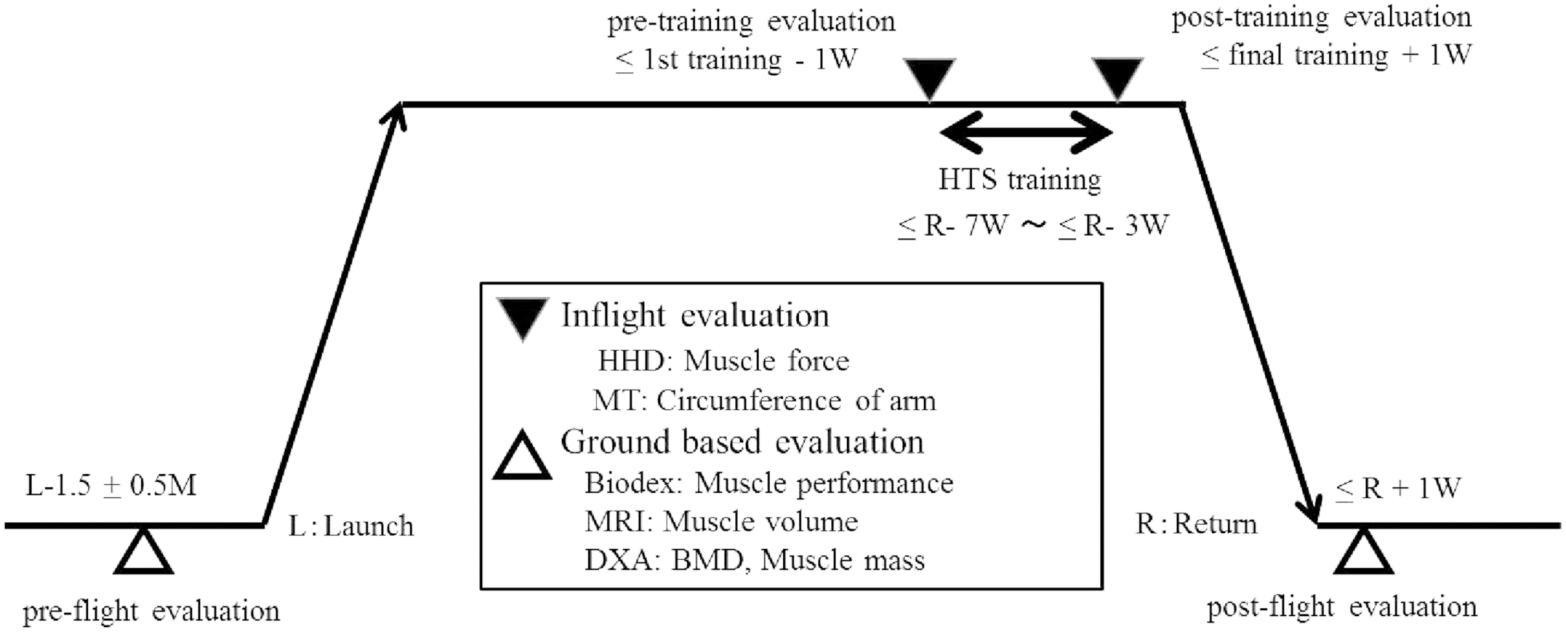
Experiment schedule. Evaluations were performed on the ground using high quality evaluation devices at pre and post flight, and inflight evaluations were also performed using simple evaluation devices within 7 days pre and post training period. Hybrid training system training took place toward the end of the flight, starting at less than R-7weeks. HHD: hand held dynamometer, MT: measuring tape, MRI: magnetic resonance imaging, DXA: dual energy X-ray absorptiometry.

HTS training took place toward the end of the flight, starting at less than return- 7weeks (Fig 3). Reciprocal elbow curls of both upper extremities, 2-seconds of flexion and 2-seconds of extension, were performed. One set of training consisted of 10 elbow curls, and 10 sets of 10 elbow curls with 1 minute rest intervals between sets were performed during a training session. 15 minutes 40 seconds were required for one session. 3 sessions were performed per week for 4 weeks, totaling in 12 sessions of training performed on the ISS.

### Evaluations

#### Ground based evaluations

Ground based evaluations were performed using high quality evaluation devices. Pre-flight evaluations were done 1.5 ± 0.5 months before launch, and post-flight evaluations were done within 7days of return to earth (Fig 3).

#### Muscle performance

Biodex System4-PRO (Biodex Medical Systems, Shirley, New York, USA) was used to evaluate muscle performance at Johnson Space Center. Measurement of elbow extension and flexion force was performed with isokinetic measurement at 180 degrees/min. In the isokinetic measurement, Peak Torque (Nm), Total Work (Joule: amount of work accomplished for the entire set) and Average Power (Watts: Total Work divided by time) were evaluated. Three sets of measurements were performed for the evaluation, and the maximum value was used for evaluation.

#### Muscle volume by MRI

Muscle volume measurement by Magnetic Resonance Imaging (MRI) (SIEMENS MAGNETOM Verio 3T, Washington, D.C., USA) was performed at the Specialty Care Center, Victory Lakes of the University of Texas Medical Branch. A 5mm diameter spherical capsule of vitamin D medicine (Alfarol, CHUGAI PHARMACEUTICAL CO. LTD., Tokyo, Japan) which contained lipid was attached as a marker on the subject’s upper arms to confirm the scan level. A scale formed from an elastic arm support band (GOLDWIN INC., Tokyo, Japan) was made to identify a constant distance from the humeral medial condyle. The arms were scanned bilaterally using T1-weighted images (TR/TE = 500/18 msWeWe). Eleven axial slices of 10mm thickness were prescribed on a coronal scan including the marker and acquired perpendicular to the main axis of the humerus. 3 slices including the center slice with the marker were used to compare muscle volume. A scale was also used for a tape measurement of arm circumference during inflight evaluation.

#### DXA

Dual Energy X-ray Absorptiometry: DXA (Hologic QDR 4500 W DXA scanner, Hologic Inc., MA, USA) was performed at Johnson Space center to evaluate bone mineral density: BMD, lean (muscle) mass and fat mass. DXA data was provided by the NASA data sharing program.

### Inflight evaluations

**Numerical Rating Scale (NRS)** was checked by the subject after the first three sessions of training to assess any pain experienced by the subject. Other inflight evaluations were performed using simple evaluation methods 6 days before and 5 days after the 4week training period (Fig 3).

#### Isometric peak force

**Fig 4:**
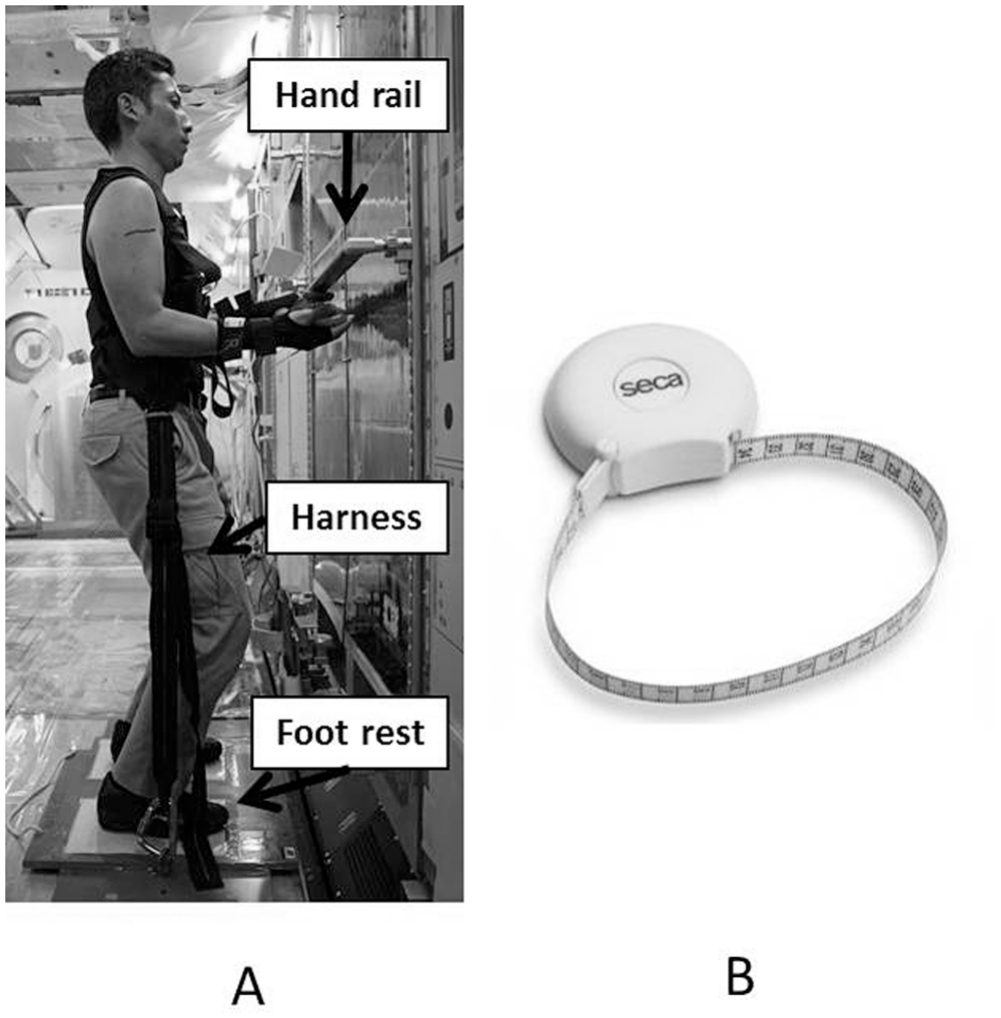
Inflight evaluations. A: A Hand Held Dynamometer: HHD is used to evaluate elbow extension and flexion strength. Elbow force measured by HHD using hand rail in erect position. Feet fixed at hand rail or foot rest. Fig 4A was photographed to explain to an astronaut at the model of the International Space Station on the ground. B: A measuring tape is used for the measurement of arm circumference.

Muscle force measurement was performed using a hand held dynamometer (HHD) (GT 310, OG Giken, Okayama, Japan) (Fig 4A). HHD was used to evaluate elbow extension and flexion strength. The subject held the HHD himself, and isometric elbow force was measured by HHD as the subject pressed it to the hand rail in an erect position with the subject’s elbow joint at 90 degrees flexion position and feet fixed in a foot rest. For the precise measurement, a wrist fixation supporter and body fixation harness were originally made and utilized. Measurement was performed three times, and the maximum value was used for evaluation.

#### Circumference of upper arm

Circumference of upper arm was measured using measuring tape: MT SECA 201 (SECA, Hamburg, Germany) (Fig 4B). The measuring point was determined at the same level as the MRI marker during the ground based evaluation utilizing the same supporter scale.

### Data analysis

All statistical calculations were performed with JMP Version 11.0 statistical software (SAS Institute Inc., Cary, NC). First, the interclass correlation coefficient (ICC) of measurements for muscle performance, muscle volume, isometric peak force, and circumference of the upper arm were calculated respectively when we measured. Next, we calculated the standard error of measurement (SEM) for each ICC. We used the minimum difference (MD) calculated by the following expression to assess the significance of the change [23, 24].

$$\text{MD }=\text{ SEM}\times 1.96\times \sqrt{2}$$

## Results

HTS training was carried out at the final phase of an astronaut’s 188-day stay on the ISS and completed 14 days before return to earth. The experiment included pre and post flight ground based evaluations and pre and post training inflight evaluations including 12 times of training which were performed on schedule. No problem occurred such as training device trouble etc. At the first training session, 35 minutes was required for preparing HTS training including stimulation intensity setting. After the first training, mean preparing and setting time for training was 8.3 ± 1.6 minutes (6–10 minutes), and mean take off and storing time was 6.1 ± 0.3 minutes (6–7 minutes).

### MDs of our measurement methods

MD of the elbow extension in peak torque/total work/average power was 0.59 (Nm/kg) /31.16 (Joule) /33.65 (Watts), respectively. MD of the elbow flexion in peak torque/total work/average power was 0.55 (Nm/kg) /26.58 (Joule) /26.94 (Watts), respectively. MD of muscle volume was 54.75 (mm^3^). MD of isometric muscle force of extension/flexion was 0.06 (N/kg)/0.09 (N/kg). MD of circumference of upper arm was 0.10 (cm).

### Ground based evaluations

#### Muscle performance (Table 1)

**Table 1:** Isokinetic measurement of Elbow extension and flexion in pre and post flight Ground Based Evaluation by Biodex, and isometric measurement peak force in pre and post training inflight evaluation by HHD.

			HTS		CTR		HTS	CTR
			pre	post	pre	post	post/pre (%)	
Extension	Ground Based Evaluation	Peak Torque (Nm/kg)	0.87	0.7	0.83	0.65	80.6	78.3
		Total Work (Joule)	217.4	199.3	214.9	215.6	91.7	100.3
		Average Power (Watts)	65.5	80	75.1	81.1	122.1	108
	Inflight Evaluation	Isometric Peak Torque (N/kg)	2.72	2.59	2.61	2.45	95.2	94.6
Flexion	Ground Based Evaluation	Peak Torque (Nm/kg)	0.49	0.38	0.44	0.44	77.6	100
		Total Work (Joule)	123	94.4	129.9	87.5	76.7	67.4
		Average Power (Watts)	37.1	34.7	35.2	33.5	93.5	95.2
	Inflight Evaluation	Isometric Peak Torque (N/kg)	2.64	2.88	2.55	2.95	109.1	115.7

HHD, hand held dynamometer; HTS, hybrid training system; CTR, control

Isokinetic elbow extension torque changed -19.4% in HTS, and -21.7% in CTR. Isokinetic elbow flexion torque changed -23.7% in HTS, and there was no change in CTR. Total Work of elbow extension changed -8.3% in HTS, and +0.3% in CTR. For elbow flexion it significantly changed -23.3% in HTS and -32.6% in CTR. Average Power of elbow extension changed +22.1% in HTS and -8.0% in CTR. For elbow flexion it changed -6.5% in HTS and -4.8% in CTR.

#### Muscle volume by MRI (Fig 5, Table 2)

**Fig 5:**
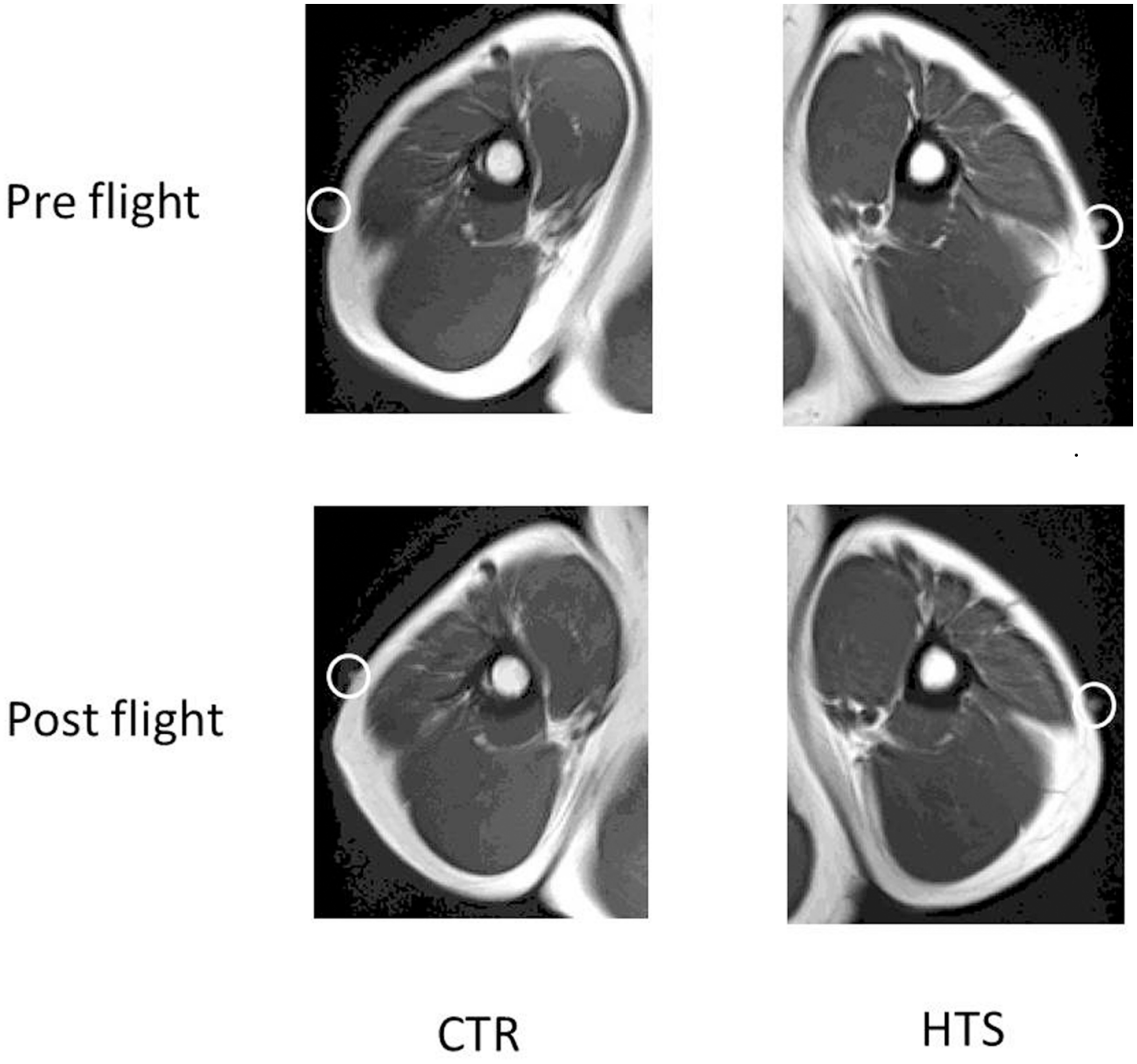
MRI findings of pre and post flight ground based evaluation. The marker was seen in each slice in a white circle.

**Table 2:** Muscle volume of upper arm using MRI in pre and post flight evaluation of Gound based evaluation, and circumference of upper arm using MT in pre and post training inflight evaluation.

		HTS		CTR		HTS	CTR
		pre	post	pre	post	post/pre (%)	
Ground Based Evaluation	Triceps muscle (mm3)	60504	67559	64599	64520	111.7	99.9
	Biceps muscle (mm3)	34320	35034	37829	37677	102.1	99.6
	Total (mm3)	94824	102594	102429	102197	108.2	99.8
Inflight Evaluation	Circumference of arm (cm)	31.5	30.7	31.4	31.2	97.5	99.4

MRI, magnetic resonance imaging; MT, measuring tape; HTS, hybrid training system; CTR, control

Volume of the triceps muscle significantly changed +11.7% and of the biceps muscle significantly changed +2.1% in HTS. In CTR, volume of the triceps significantly changed -0.1% and of the biceps also significantly changed -0.4% in CTR.

### DXA (Table 3)

**Table 3:** DXA of BMD, lean (muscle) mass and fat mass in upper extremities in Ground Based Evaluation.

	HTS		CTR		HTS	CTR
	pre	post	pre	post	post/pre (%)	
BMD (g/cm2)	0.786	0.822	0.848	0.833	104.6	98.8
Lean (muscle) (g)	2979	3294	3087	3087	110.6	100
Fat (g)	21	18	21	20	87.4	93.6

DXA, dual energy x-ray absorptiometry; BMD, bone mineral density.

Data was provided by data shearing program of NASA.

According to the data share of DXA with NASA, BMD changed +4.6% for the upper arm using HTS, however it changed -1.2% for CTR. Muscle mass changed +10.6% for the upper arm using HTS, but there was no change for CTR. Fat mass in the upper arm changed -12.6% for HTS and -6.4% for CTR.

### Inflight evaluations

**NRS** showed 0, indicating that the subject experienced no pain during any of the exercise sessions. Isometric elbow extension force significantly changed -4.8% for HTS and -0.8% for CTR. Isomeric elbow flexion force significantly changed +9.1% for HTS and +15.7% for CTR (Table 1).

#### Circumference of upper arm

According to inflight tape measurement, circumference of the upper arm significantly changed -2.5% for HTS and -0.6% for CTR (Table 2).

## Discussion

In the current experiment, MRI and DXA results showed that muscle volume, mass and BMD had increased in an astronaut’s upper arm after completion of 4 weeks of HTS training. HTS functioned well without problems and demonstrated a high level of orbital operation capability and utility, as well as the possibility in preventive effect by HTS for musculoskeletal atrophy of astronauts on the ISS. The present experiment was performed by an astronaut as the subject. It will not be feasible to generate large enough sets of data to allow inferential statistics, and this is a common limitation of in-flight experiments on the ISS with human participants due to operational restrictions and the small number of crew members. Because the experiment was designed to verify the training device for astronauts, an actual astronaut as a subject was essential.

The upper extremity was selected for the current experiment so that it would not affect ISS operation or interfere with other physical condition experiments on the ISS. To make the current space experiment possible, it was necessary to select the upper arm for the initial verification of HTS on the ISS. However, musculoskeletal atrophy and functional deterioration are remarkable in lower extremities among astronauts who stay on the ISS for a long term [1, 2], therefore countermeasures for the lower extremities are clearly more important than for the upper extremities. Future experiments involving lower extremities are essential to verify the effects of HTS in weightless conditions such as on the ISS. Furthermore exercise effect is easier to obtain, in percentage terms, muscle hypertrophy in non-weight-bearing muscles of the neck and upper limbs [25–27]. When used in lower extremities, the training effect of the HTS may be different.

HTS was developed as a countermeasure for the musculoskeletal atrophy of astronauts and uses an eccentric contraction produced in a muscle by electrical stimulation of the antagonist to resist the volitional concentric contraction of the agonist. In other words, electrical stimulation generates a resistive force instead of gravity. HTS has several beneficial features. These include; 1) a simple, small device, 2) requiring minimal external stabilization of the subjects, 3) simultaneous contractions of both agonist and antagonist muscles, 4) volitional contraction of deep layers of muscle and 5) longitudinal bone force loads.

In the present experiment, approximately 30 minutes was required for a training session of HTS including preparation time (8.3 ± 1.6 minutes), training time (15 minutes 40 seconds), and storing time (6.1 ± 0.3 minutes) excepting the first training session. HTS was compact and its setting and storage were easy enough to use in weightlessness such as on the ISS. Electrodes installed inside the supporter enabled an accurate setting position and effectively shortened the installation time in weightless conditions. Because the power supply of the HTS device is battery which is already available in orbit, it is portable.

Increase in muscle volume is considered an effect of simultaneous muscle contractions; such as reciprocal electrical simulated eccentric contraction and voluntary concentric contraction of muscles [16–18]. In the current experiment, the average elbow flexion total work decreased by 23.3% in HTS, which was less than the 32.6% in CTR, according to ground based isokinetic muscle performance evaluation. Total work means the amount of work accomplished for the entire set, and it is used to measure muscle endurance. Decrease in muscular endurance is one of the indications of musculoskeletal system disuse among astronauts. HTS might prevent decrease in muscular endurance. However the other muscle force measurement results from ground based evaluations in this study didn’t show an increase A significant efficacy of HTS was not observed in inflight evaluations either, but the statistical comparison test of these muscle force measurements will be altered by increasing the number of subjects clinically because of the possibility of individual differences. In this experiment, however, we were forced to evaluate a single subject due to limitation of resources for a space experiment. Increasing the number of measurements might enhance its reliability even for a single subject, though this was difficult in the case of our experiment because of restrictions in crew time.

A tape measure was used for inflight evaluation due to restrictions in carrying-on high functional evaluation devices for this ISS experiment. Circumferences of both upper arms were measured, and both of them had decreased. Decrease in HTS circumference was greater than for CTR, even though muscle mass had increased according to the ground based MRI and DXA tests. In the DXA study, decrease of fat mass was greater in HTS than that in CTR, and this was considered to be one of the reasons for the decrease in circumference for HTS as expressed by measuring tape.

The increase in BMD is considered to be the effect of longitudinal bone force loads from HTS. HTS training applied longitudinal force load on the bone by contraction of sandwiched muscles simultaneously. Theoretically the longitudinal force load on the bone is the total muscle force of the agonist and antagonist, and it means that roughly their total muscle force is obtained as a longitudinal load force on the long bone as opposed to usual exercise. The bone was exposed to 200 instances of this load per training, consisting of 100 elbow curls. This mechanical stress was loaded for every flexion and extension during elbow curls repeatedly, and might have increased BMD effectively.

BMD increased by 4.6% in the HTS arm, and its increase ratio appears to be greater compared to the other space flight experiments such as using Bisphosphonate [2, 11]. Astronauts are exposed to absolute unloading conditions in the ISS except for their 2.5 hours/day exercise time. An astronaut, who didn’t take Bisphosphonate during this flight, used HTS for 15 minutes 40 seconds a day, 3 times a week for 4 weeks, and the repetitive longitudinal load force might have effectively provided a mechanical stress on the long bone and increased BMD in this experiment. HTS successively supplemented the training effects on the arm of an astronaut over and above his regular 2.5 hours of daily comprehensive exercise, and the results may have become more evident due to the absolute unloading condition on the ISS as opposed to those on the ground.

HTS utilizes electrical eccentric muscle contractions, which provide 30–50% greater force even when using identical stimulation intensity to that of concentric or isometric contractions [28–32]. Therefore HTS can be used at a much lower stimulation intensity than that of conventional neuro muscular electrical stimulation for training, this would reduce the galvanic pain and possible deterioration caused by electrical stimulation. Threshold and the maximal comfortable stimulation intensities were examined as in past experiments on the ground [16–22]. Electrical stimulation intensity was set at less than 30% of the maximal voluntary contraction, and at less than or equal to 80% of the maximal comfortable stimulation as in past multiple ground based experiments [16–22]. The maximum current of stimulation intensity applied to subjects during optimization does not exceed 20mA in the current experiment, and a constant stimulation intensity was used throughout the entire 4 week training period. This was 21.0V on the triceps and 15.5V on the biceps muscles respectively in the present experiment on the ISS.

HTS has already been shown to be both technically sound and clinically effective in multiple ground-based experiments [16–22]. More specifically, the method is capable of increasing torque production and muscle mass in the upper [16] and lower extremities by amounts that are comparable to that of conventional weight training [17]. In 4 patients with knee anterior cruciate ligament: ACL injury, HTS was carried out preoperative for 4 weeks [18]. Gene ontology analysis revealed that HTS induced significantly higher expressions of the factor which enhances muscle regeneration through its role in satellite cell differentiation and a prerequisite factor which plays an important role in muscle regeneration after injury. In this study, these characteristic effects of HTS might result in muscle hypertrophy and increasing total work. HTS showed not only a musculoskeletal system effect but also a systemic effect. HTS decreased fasting blood glucose and serum interleukin-6: IL-6 levels in elderly people [19]. HTS also reduces steatosis, insulin resistance, and IL-6 levels in patients with non-alcohol fatty liver disease [20]. In this study, this effect to lipid metabolism of HTS might have decreased fat.

As stay on the ISS becomes more extended, the problem of the astronaut’s musculoskeletal atrophy becomes important. A focus on the development of a countermeasure has been to give load to the trunk and lower limbs which was the main site of atrophy and bone loss. A passive "treadmill" using rubber bungee cords or the MK-I and MK-II” Mini Gym" using springs were used on Skylab. The Skylab 4 crewmembers who used the MK-I and MK-II”Mini Gym", as well as a passive "treadmill" showed suppressed muscle strength weakness [33]. Because the ISS is large, astronauts can use these large countermeasures which can give a bigger load. ARED was developed to overcome the disadvantages of exercise with elastic bands such as rubber bungee cords which cannot provide a consistent large load during exercise. ARED appears to be eliciting better strength maintenance than previous countermeasures [15]. However exercise equipment failures and other constrains have limited the access of ISS crewmembers to the full complement of aerobic and resistance exercise protocols [15]. Thus, HTS may be used as substitute method for ARED although it may be not as effective. Furthermore, HTS could be available on a small spaceship which will be indispensable for missions to the moon, establishment of a lunar base, and interplanetary travel to Mars, because HTS is a small, lightweight, and portable training device.

A future HTS experiment of the lower extremity should be performed in weightlessness to prove its actual orbital effectiveness as mentioned above. HTS can be used with other exercise or training, and we tested it with an ergometer [21]. HTS was able to be used easily with the ergometer and functioned well. HTS provided the advantages of both resistance exercise by electrical eccentric contractions and aerobic exercise at low exercise intensity by voluntary concentric contractions. It was considered that HTS may improve physical ability also. HTS could be used together with other training and serve to shorten training time and thus save the crew time.

## Conclusions

Results of the present experiment showed the orbital operation capability and utility, and the possibility in preventive effect of HTS on an astronaut’s musculoskeletal atrophy, even though HTS should be used on the lower extremity in the future. HTS is the only small and lightweight training device developed to date that can provide an effective exercise load in weightlessness. The initial flight data together with the ground data obtained so far will be brought to future planning of human space exploration. HTS may be useful in keeping astronauts fit as they travel beyond low Earth orbit aboard smaller spacecraft.
