# Correction: Electrically Stimulated Antagonist Muscle Contraction Increased Muscle Mass and Bone Mineral Density of One Astronaut - Initial Verification on the International Space Station

**DOI:** 10.1371/journal.pone.0138519

**Published:** 2015-09-14

**Authors:** Naoto Shiba, Hiroo Matsuse, Yoshio Takano, Kazuhiro Yoshimitsu, Masayuki Omoto, Ryuki Hashida, Yoshihiko Tagawa, Tomohisa Inada, Shin Yamada, Hiroshi Ohshima

There is an error in the affiliation 5 and the current address for authors Shin Yamada and Hiroshi Ohshima. Affiliation 5 should be: Space Biomedical Research Group, Japan Aerospace Exploration Agency, Tsukuba, Ibalaki, Japan. The current address should read: Space Biomedical Research Group, Japan Aerospace Exploration Agency, 2-1-1, Sengen, Tsukuba, Ibaraki 3058505, Japan.
